# Current issues and perspectives in PD-1 blockade cancer immunotherapy

**DOI:** 10.1007/s10147-019-01588-7

**Published:** 2020-01-03

**Authors:** Kenji Chamoto, Ryusuke Hatae, Tasuku Honjo

**Affiliations:** Department of Immunology and Genomic Medicine, Kyoto University Graduate School of Medicine, Yoshida Konoe-cho, Sakyo-ku, Kyoto, 606-8501 Japan

**Keywords:** Immune checkpoint inhibitor, Biomarker, Immune-related adverse event, Immune metabolism, Combination therapy

## Abstract

Programmed cell death 1 (PD-1) signal receptor blockade has revolutionized the field of cancer therapy. Despite their considerable potential for treating certain cancers, drugs targeting PD-1 still present two main drawbacks: the substantial number of unresponsive patients and/or patients showing recurrences, and side effects associated with the autoimmune response. These drawbacks highlight the need for further investigation of the mechanisms underlying the therapeutic effects, as well as the need to develop novel biomarkers to predict the lack of treatment response and to monitor potential adverse events. Combination therapy is a promising approach to improve the efficacy of PD-1 blockade therapy. Considering the increasing number of patients with cancer worldwide, solving the above issues is central to the field of cancer immunotherapy. In this review, we discuss these issues and clinical perspectives associated with PD-1 blockade cancer immunotherapy.

## History of cancer immunology: basic to clinical research

The hypothesis that most cancer cells are eliminated by the host immune system during cancer development is known as “immunological surveillance”; this concept was proposed by Burnet in the 1960s. However, this theory failed to explain the reasons for cancer proliferation despite immunological surveillance. In 2002, Dunn et al. referred to “cancer immunoediting”, which comprises three stages: the “elimination phase”, “equilibrium phase”, and “escape phase” [1]. In the elimination phase, abnormally proliferating cells are eliminated via immunological surveillance. Abnormally proliferating cells that are not eliminated enter the precancerous, equilibrium phase in which apparent tumor size remains unchanged, as the rate of elimination by immune cells is equal to the proliferation rate of abnormal cells. However, once abnormal cells have acquired mechanisms that allow them to escape immunological surveillance, they enter the escape phase, where a growing mass is recognized as cancer. Anti-tumor immunity involves various immune cells, but the final effector cells are cytotoxic T lymphocytes (CTLs), which specifically recognize and kill diverse antigens expressed on cancer cells. Cancer immune escape involves the loss of tumor antigen, human leukocyte antigen (HLA), and expression of immunosuppressive molecules in tumor cells, as well as induction of immunosuppressive cells.

**Fig. 1:**
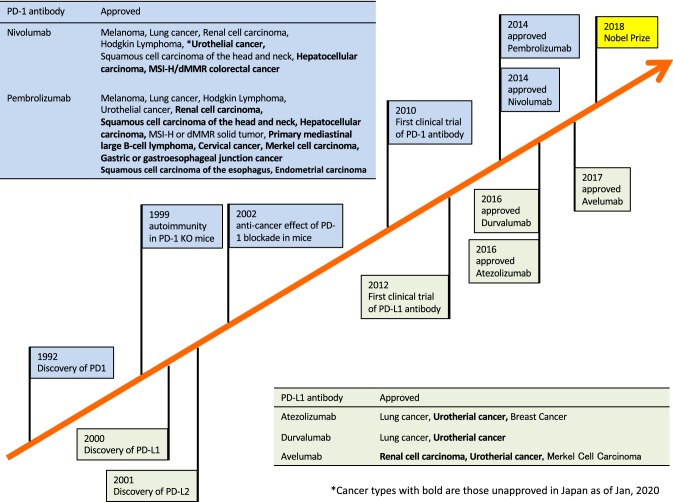
History of programmed cell death-1 (PD-1) blockade cancer immunotherapy development

Programmed cell death 1 (PD-1) is an immunosuppressive co-stimulatory signal receptor that belongs to the CD28 family. PD-1 was first identified by Ishida et al*.* in 1992 as a programmed cell death-induced gene encoding type I membrane proteins in T cells (Fig. 1) [2]. PD-1 was shown to be an immune suppressive factor based on the development of autoimmune diseases in PD-1 receptor-deficient mice [3-6]. PD-1 is expressed primarily on activated T cells and B cells, and serve as an immune regulator that controls inappropriate and extreme immune responses such as autoimmune and excessive infectious immune responses. It suppresses antigen receptor activation by PD-ligand 1 (PD-L1) and PD-L2, which belong to the co-stimulatory signal B7 family. Although PD-L1 is widely expressed on antigen-presenting cells (dendritic cells, blood vessels, myocardium, lung, and placenta), PD-L2 is present on dendritic cells and is only expressed in activated macrophages. Binding of PD-1 to PD-L1/2 is primarily related to immunosuppression in the peripheral tissue. Indeed, PD-L1-introduced tumors grow quickly in wild-type mice but not in PD-1-knockout mice, indicating that PD-1 plays a central role in cancer cell immune escape mechanisms [7]. Based on the hypothesis that interruption of anti-PD-1/PD-L1 binding may activate T cells against cancer cells, PD-1 blocking antibodies have been developed as immune checkpoint inhibitors for cancer therapy [7] (Fig. 1).

The clinical efficacy of nivolumab, an antibody against human PD-1, was subsequently reported in 2010 and 2012 [8, 9]. In 2014, nivolumab was approved in Japan for the treatment of malignant melanoma for the first time worldwide. PD-1/PD-L1 antibody-based therapy is currently approved for the treatment of various cancers (Fig. 1). However, more than half of patients do not respond to this therapy [10].

Improving the response rate in patients with cancer relies on three different approaches: (1) elucidating the mechanisms underlying the lack of response to PD-1 antibody treatment, (2) developing novel predictive markers, and (3) developing an effective combination therapy. These approaches and the status of current research are discussed in the subsequent sections.

## Biomarkers

**Fig. 2:**
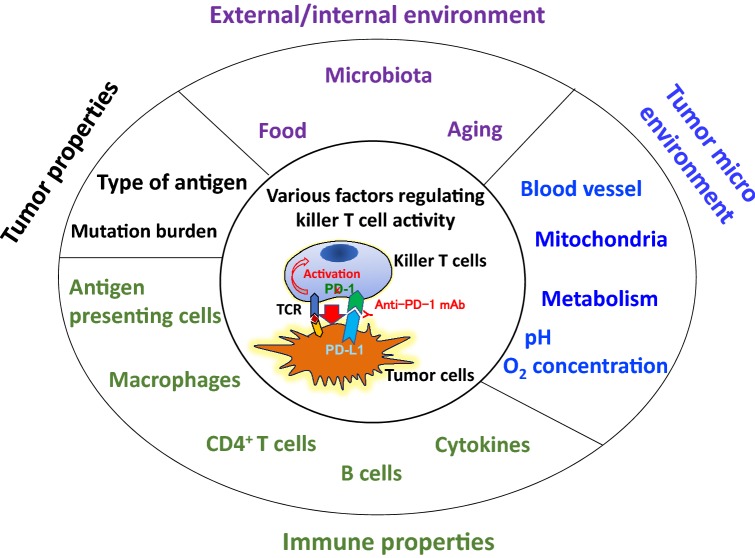
Regulation of killer T cell activity by various factors during PD-1 blockade therapy

Killer T cells are the final effector immune cells that attack cancer cells. Killer T cell activity cannot be predicted by any single biomarker as it is controlled by various factors (Fig. 2), including tumor- and immune-related factors, as well as environmental factors such as enterobacteria and metabolism.

### Biomarkers-tumor-related factors

A nivolumab phase I clinical study revealed that PD-L1 expression in tumor cells may be an indicator of treatment efficacy [8]. Several clinical trials subsequently evaluated whether PD-L1 expression could be a predictive biomarker. However, a significant association between improved outcomes and PD-L1 expression was observed only in certain cancers [11]. According to clinical studies showing a positive association, the United States (US) Food and Drug Administration (FDA) approved pembrolizumab for the treatment of PD-L1-positive non-small cell lung cancer (NSCLC), gastric or gastroesophageal junction cancer, and cervical cancer in 2015, 2017, and 2018, respectively. Furthermore, considering IMpassion130 study results, the FDA approved therapy with atezolizumab (a PD-L1 antibody) and chemotherapy (nab-paclitaxel) for PD-L1-positive and metastatic triple-negative breast cancer (TNBC) in March 2019 [12].

Two main mechanisms are hypothesized to be involved in PD-L1 expression in tumors: forced expression of PD-L1 due to translocations or mutations [13], and stimulation of intra-tumoral T cell-produced interferon, also known as “adaptive resistance” [14-16]. Because adaptive resistance is regulated by immune cell activity, PD-L1 expression show a correlation with PD-1 blockade therapy prognosis. However, insufficiency of tumor PD-L1 expression as a biomarker may result from difficulties in distinguishing the above two mechanisms. Moreover, PD-1/PD-L1 expression in tumor-infiltrating immune cells (T cells and macrophages) is reported to be involved in the therapeutic effects in malignant melanoma or bladder cancer [15, 17-19].

PD-1 antibodies are effective for tumors with somatic mutations, such as malignant melanoma, lung cancer, and renal cell carcinoma (RCC) [20]. Tumor-infiltrating T cells recognize mutated peptides as foreign antigens (neoantigens), thus inducing a strong immune response. Rizvi et al*.* demonstrated a significant correlation between therapeutic effect and neoantigen number, DNA repair pathway mutations, and non-synonymous mutations in pembrolizumab-treated patients with NSCLC [21]. In the CheckMate 026 study (phase III), which used nivolumab as first-line NSCLC treatment, high tumor mutational burden (TMB) tumors were likely to show greater responses [22]. However, another study reported a partial response even in low-TMB RCC [23]. This suggests the importance of the mutation type in addition to the number, as RCC frequently contains indel mutations (DNA insertion and deletion), which produce frameshift and variety of neo-antigens [23]. Future studies are needed to measure and evaluate the type and number of mutation, which affect the efficacy.

A phase II study evaluated the effect of pembrolizumab (NCT01876511) in colorectal cancer with deficiency of DNA mismatch repair (MMR) ability (dMMR), those with functional MMR protein expression, and all solid tumors with dMMR. Therapeutic efficacy was low in colorectal cancer with MMR protein expression, but was high in all solid tumors with dMMR [24]. Whole-exome sequencing showed that the average number of somatic mutations per tumor with dMMR and tumors with functional MMR (1782 and 73, respectively) was significantly correlated with therapeutic effect. Moreover, the number of neoantigen-specific T cells in responding patients was significantly higher than that in non-responders in the NCT01876511 study. Accordingly, the FDA approved pembrolizumab in May 2017 for unresectable/metastatic solid cancers with high microsatellite instability (MSI-H) or dMMR in adults and children. Furthermore, the FDA approved nivolumab and the combination of nivolumab and ipilimumab in August 2017 and in July 2018, respectively, for MSI-H or dMMR metastatic colorectal cancer [25]. It is the first time that a treatment has been approved based on the biomarker rather than tumor type [26].

### Biomarkers-immunity-related factors

Higher numbers of tumor-infiltrating CTLs are correlated with better prognosis. This is known as the “immunoscore” [27, 28], in which CD8^+^ T cell quantification at the tumor center and periphery can strongly predict the overall survival (OS) and well correlated with the traditional tumor-node-metastasis (TNM) staging and/or MSI status in patients with colorectal cancer [28-30]. Moreover, the number of CD8^+^ T cells around the tumor was correlated with therapeutic effect in PD-1 antibody-treated patients with malignant melanoma treated [16]. The number of infiltrated CD8^+^ T cells was also correlated with high PD-L1 expression in tumor cells and the therapeutic effect of PD-1 antibodies [31]. However, the immunoscore is not a perfect marker as the significance of CTL tumor infiltration may vary depending on the type of carcinoma. For instance, in the case of RCC, higher numbers of tumor-infiltrating CD8^+^ T cells are correlated with poor prognosis [32].

Since tumor tissue biopsy may be extremely invasive, the use of less invasive biomarkers, such as those in peripheral blood, would represent an ideal approach. High neutrophil/lymphocyte ratio (NLR), which is typically measured in conventional blood tests, is associated with poor prognosis in cytotoxic T lymphocyte antigen-4 (CTLA-4) antibody-treated patients with malignant melanoma [33] and in PD-1 antibody-treated patients with various cancer [34, 35]. CD4^+^ T cells increase in the periphery is reported to be related to good response after nivolumab treatment [36, 37]. Cells release extracellular exosomes containing their surface molecules [38, 39]. While an increase in circulating exosomal PD-L1 in the blood before anti-PD-1antibody treatment was associated with poor response in patients with melanoma [38], increased PD-1 and CD28 expression in exosomes, which may be derived from T cells, was associated with improved progression-free survival (PFS) in anti-CTLA4-treated patients [40]. Instead of many ongoing researches, no peripheral blood biomarker for immune-related factors has been approved by the FDA for clinical use.

### Biomarkers-microbiota

More than 1000 microorganism species and 100 trillion bacteria coexist in the human body. The maturation and barrier function of the immune system were significantly impaired in germ-free and antibiotic-treated mice. This immune impairment is normalized by transplanting mouse intestinal bacteria. The intestinal microbiota produces metabolites (such as short-chain fatty acids) from indigestible polysaccharides. The short-chain fatty acid butyric acid regulates immunity and metabolism by binding to G-protein-coupled receptors and promoting epigenome modifications [41]. A significant correlation between PD-1 blocking treatment efficacy and enterobacteria (i.e., *Bifidobacterium*) levels was reported in a mouse model of melanoma B16 cells, suggesting the potential of microbiota composition as a predictive biomarker for PD-1 blockade therapy [42].

Routy et al*.* reported the effects of antibiotics on the response to immune checkpoint inhibitors in patients with NSCLC, RCC, and urothelial carcinoma [43]. PFS and OS significantly decreased after PD-1/PD-L1 antibody treatment in patients with a history of antibiotic treatment compared to those in patients without. Intestinal microbiota damage caused by antibiotics may therefore attenuate the anti-tumor immune response to immune checkpoint inhibitors. Moreover, intestinal microbiota composition differed between PD-1 antibody treatment responders and non-responders, and patients with a high proportion of *Akkermansia muciniphila* species in their microbiome showed better treatment outcomes. A significant correlation between type 1 T helper (Th1) responses to bacteria and treatment outcome has also been demonstrated [43]. Gopalakrishnan et al*.* and Matoson et al*.* investigated intestinal microbiota composition in PD-1 antibody-treated patients and found a significant correlation between enterobacteria diversity and responsiveness to treatment [44, 45]. Furthermore, a recent study reported that 11 types of enterobacteria isolated from healthy individuals’ feces led to CD8^+^ T cell activation, and that mice inoculated with these bacteria species showed greater tolerance to infection and anti-cancer immune responses [46]. Overall, studying crosstalk between the intestinal microbiome and the immune system would be useful for both potential biomarker candidate discovery and effective combination therapy development.

## Side effects

Immune checkpoint inhibitors may cause fewer adverse events than conventional chemotherapy (Table 1) [10, 47-49]. It is of note that PD-1 antibodies generate less severe side effects than CTLA-4 antibodies [50, 51]. Combination therapy with PD-1 antibodies and chemotherapy showed side effects similar to those of chemotherapy alone, whereas combination therapy with PD-1 and CTLA-4 antibodies may cause side effects more severe than those associated with either monotherapy (Table 1) [51-53]. Immune checkpoint inhibitor treatment requires precautions because of potential immune-related adverse events (irAEs), which differ from the adverse events observed during conventional chemotherapy. irAEs include rash with itching, diarrhea, enteritis, hepatitis, hypophysitis, thyroiditis, pneumonitis, type 1 diabetes, myositis, peripheral neuritis, and myasthenia gravis. Although many of the side effects are mild, reversible, and easy to treat, it is important to be aware of potential sever irAEs and corresponding treatment.

**Table 1:** Grade 3–5 adverse event related with treatment in phase III clinical trials of PD-1 antibody

Clinical study	Tumor	Drugs	Cases	Incidence of grade 3–5 adverse event related with treatment (%)
CheckMate 066 [10]	Untreated metastatic melanoma without BRAF mutations	Nivolumab	206	11.7
		Dacarbazine	205	17.6
KEYNOTE-006 [50]	Advanced melanoma	Pembrolizumab every 2 week	278	13.3
		Pembrolizumab every 3 week	277	10.1
		Ipilimumab	256	19.9
CheckMate 067 [51]	Untreated stage III or IV melanoma	Nivolumab alone	313	16.3
		Ipilimumab alone	311	27.3
		Nivolumab plus ipilimumab	313	55.0
CheckMate 017 [47]	Advanced squamous-cell NSCLC	Nivolumab	131	7
		Docetaxel	129	55
CheckMate 057 [48]	Advanced non-squamous NSCLC	Nivolumab	287	10
		Docetaxel	268	54
KEYNOTE-189 [49]	Previously treated NSCLC with PD-L1 expression on at least 1% of tumor cells	Pembrolizumab 2 mg/kg	339	13
		Pembrolizumab 10 mg/kg	343	16
		Docetaxel	309	35
KEYNOTE-189 [52]	Metastatic non-squamous NSCLC without sensitizing EGFR or ALK mutations	Pembrolizumab plus chemotherapy^b^	410	67.2
		Chemotherapy^b^	206	65.8
KEYNOTE-407 [53]	Untreated metastatic, squamous-cell NSCLC cancer	Pembrolizumab plus chemotherapy^c^	278	69.8
		Chemotherapy^c^	281	68.2

*NSCLC* non-small cell lung cancer, *PD-L1* programmed cell death ligand 1

^a^Original sources are given as reference numbers

^b^Four cycles of the investigator’s choice of intravenously administered cisplatin 75 mg/m^2^ or carboplatin (area under the concentration–time curve, 5 mg/mL/min) plus pemetrexed (500 mg/m^2^), all administered intravenously every 3 weeks, followed by pemetrexed 500 mg/m^2^ every 3 weeks

^c^Carboplatin (at a dose calculated to obtain an area under the concentration–time curve of 6 mg/mL/min) on day 1 and either paclitaxel 200 mg/m^2^ on day 1 or nab-paclitaxel 100 mg/m^2^ on days 1, 8, and 15

Treatment for irAEs relies on diagnosis to exclude non-inflammatory diseases, followed by a treatment to reduce symptoms, steroid treatments, or administration of the anti-tumor necrosis factor (TNF)-*α* antibody infliximab depending on symptom severity [10, 54].

Although most side effects are not severe, they often include asymptomatic endocrine consequences. Early detection and intervention are essential to reduce the risk of irAE-related side effects. Steroid treatment for irAEs has been reported to improve symptoms, although in some cases, anti-tumor immunity persists [55, 56], perhaps because of stronger immune responses to cancer antigens than to autoantigens. Therefore, in certain patients steroids do not sufficiently suppress immune responses to tumors, but is enough to inhibit autoimmune responses. Further studies are required to monitor autoimmune responses without impairing anti-tumor immunity and develop biomarkers that predict fatal side effects.

## Tolerance to PD-1 blockade therapy

Although PD-1/PD-L1-blocking antibodies have longer therapeutic effects than conventional chemotherapy, resistance may occur. Zaretsky et al*.* analyzed biopsy specimens from four patients who first experienced tumor reduction prior to progression several years after pembrolizumab treatment. Whole-exome sequencing was performed on pairs of biopsy specimens before and after the treatment and revealed interferon receptor-related Janus kinase (JAK)1/JAK2 mutations in two of four patients, as well as mutations of histocompatibility complex (MHC) class I expression in another patient [57]. Shin et al*.* showed that malignant melanoma and dMMR colorectal cancer with JAK1/JAK2 mutations led to initial pembrolizumab treatment resistance despite the high number of somatic mutations. In the study, they demonstrated that JAK function loss, which inactivates the interferon signaling pathway, is involved in the impairment of antigen presentation in tumor cells [58]. Anagnostou et al*.* analyzed four patients with NSCLC who became refractory after receiving combination therapy with PD-1 and CTLA-4 antibodies. Mutation-related neoantigens were reduced in size after resistance acquisition and were involved in resistance to checkpoint inhibitors [59]. Gong et al*.* performed tumor gene analysis in 17 patients who had acquired tolerance to PD-L1 antibodies and identified secreted RNA splicing of PD-L1 protein variants in four patients, which competitively neutralized anti-PD-L1 antibody activity [60]. Although all analyses were performed on a relatively small sample, these findings promote the development of effective combination therapies such as immune checkpoint inhibitors.

## Combination therapy

### Combination with chemotherapy

The effects of typical cytotoxic anticancer drugs on anti-tumor immune responses are well known. Combination may improve the effect of immune checkpoint inhibitors via dendritic cell activation with increased neoantigen presentation, MHC upregulation, and inhibition of immunosuppressive cell activity [including regulator T cells (Tregs), M2 macrophages, and myeloid-derived suppressor cells (MDSC)] [61-63]. In the KEYNOTE-021 clinical study (phase II), patients with PD-L1-positive non-squamous NSCLC without epidermal growth factor receptor (EGFR) mutations or anaplastic lymphoma kinase (ALK) genetic translocation were tested with pembrolizumab and chemotherapy (carboplatin and pemetrexed). Patients were divided into three groups: pembrolizumab and chemotherapy group (combination therapy), maintenance therapy group, and chemotherapy only group. The response rates were 29% and 55% in the chemotherapy alone and combination therapy groups, respectively. Accordingly, the FDA approved combination therapy with pembrolizumab and chemotherapy for NSCLC in May 2017. In the KEYNOTE-189 and KEYNOTE-407 studies (phase III), PFS and OS were significantly longer in patients treated with pembrolizumab and chemotherapy than that in patients treated with chemotherapy alone. The FDA therefore extended indications for pembrolizumab and chemotherapy combination therapy [52, 53]. Moreover, the IMpower150 study (phase III) showed that PFS was longer in the combination therapy group (with chemotherapy/angiogenesis inhibitor bevacizumab), resulting in FDA approval for NSCLC in December 2018 [64]. In the IMpower133 study (phase III), atezolizumab and chemotherapy (carboplatin and etoposide) significantly prolonged both PFS and OS in patients with extensive stage small cell lung cancer, resulting in FDA approval of this combination as first-line therapy in March 2019 [65]. In addition, in the IMpassion130 study (phase III), atezolizumab and albumin-bound paclitaxel resulted in significantly longer PFS than that in the chemotherapy only group, leading to FDA approval for TNBC in March 2019 [12]. It is well known that angiogenesis supports the tumor growth. Vascular endothelial growth factor (VEGF) inhibitor inhibits the tumor growth by attenuating the angiogenesis or normalized aberrant vessel structure [66]. It is noticeable that VEGF receptor tyrosine kinase inhibitor, Axitinib combined with PD-1 blockade has improved the survival of patients with advanced renal-cell carcinoma and approved by FDA though the precise mechanisms remains unknown [67, 68].

Overall, these clinical studies support the enhanced efficacy of combination therapy with chemotherapy and immune checkpoint inhibitors. Considering that monotherapy with immune checkpoint inhibitors also showed significant therapeutic effects in a substantial subset of patients, the development of novel biomarkers is necessary to distinguish patients requiring only monotherapy from those requiring combination therapy.

### Combination with CTLA-4 antibodies

Other immune checkpoint molecules, such as CTLA-4, T cell immunoglobulin mucin 3 (Tim3), and lymphocyte activation gene 3 (LAG3), function as immune brakes and blocking antibodies for these molecules are therefore called immune checkpoint inhibitors. CTLA-4 antibodies were first developed in the 1990s [69]. A clinical trial reported that patients treated with the CTLA-4 antibody ipilimumab showed good treatment outcomes. In 2011, the FDA approved CTLA-4 antibodies as therapeutic agents for malignant melanoma. Combining the therapeutic effects of PD-1 and CTLA-4 blockade appears logical because of their different targets and mechanisms of action. Dendritic cells activate CTLs by presenting tumor antigens in lymph nodes, leading to the elimination of cancer cells expressing the same antigens. In the early phase of T cell priming, CD28 located on T cells were bound to B7 (CD80 and CD86) on dendritic cells in addition to the interaction between T cell receptor and MHC. CTLs prevent excess activation via the B7 receptor CTLA-4, which binds to B7 with approximately 20 times more affinity than CD28, resulting in the CD28 signal blockade. CTLA-4 antibodies recover T cell activation by blocking the competition with CD28 [70]. CTLA-4 antibodies enhance anti-tumor activity by suppressing Treg activity or reducing the number of Tregs in the tumor tissue via antibody-dependent cellular cytotoxicity (ADCC) due to CTLA-4 overexpression on Tregs [71-73]. However, precise mechanisms remain unclear.

In the CheckMate 069 (phase II) and CheckMate 067 (phase III) studies for malignant melanoma patients, combination therapy with ipilimumab (human CTLA-4 antibody) and nivolumab significantly prolonged PFS compared with each alone, resulting in FDA approval of this combination therapy [51, 74]. However, the incidence of irAEs was relatively high (Table 1). The long-term follow-up data in the CheckMate-067 study revealed that the frequency of the patients who required discontinuation of the therapy due to severe irAEs was 30%, 8%, and 40% in the ipilimumab-nivolumab combination therapy, nivolumab treatment, and ipilimumab treatment, respectively. Moreover, the incidence of side effects of ≥ grade 3 was 59%, 21%, and 28%, respectively [75]. In the CheckMate 214 study (phase III) of RCC, which compared combination therapy with ipilimumab-nivolumab and standard treatment, the combination therapy group showed significantly longer OS and higher response rate [76]. However, certain patients in the sunitinib group who were classified as patients with “favorable risk” (good prognosis) according to the International Metastatic Renal Cell Carcinoma Database Consortium (IMDC) risk classification showed better outcomes than patients with the combination therapy. Therefore, in April 2018, the FDA approved combination therapy with ipilimumab and nivolumab for intermediate-/poor-risk patients (poor prognosis) in IMDC classification with untreated advanced RCC [76].

The efficacy of combination therapy with ipilimumab and nivolumab in patients with NSCLC was confirmed by the CheckMate-227 study [77]. In this study including patients with high TMB, the ipilimumab-nivolumab combination therapy group showed significantly longer PFS than the standard treatment group [77]. Treatment with a combination of PD-1 and CTLA-4 antibodies shows promise if irAEs are well controlled and if predictive biomarkers for irAEs are developed.

### Improvement of cancer immunotherapy via metabolic control

**Fig. 3:**
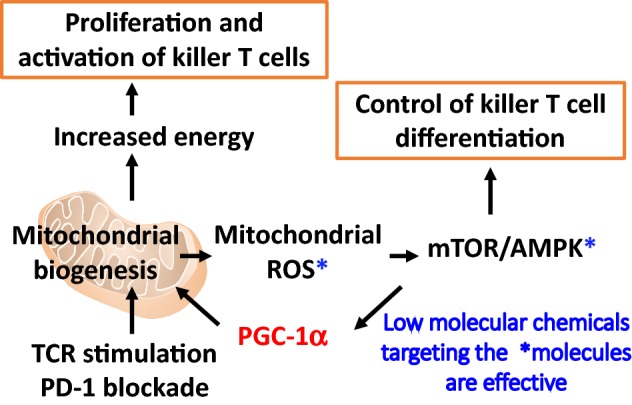
Low-molecular weight drugs activating reactive oxygen species (ROS) production mTOR/AMPK, or PPAR gamma coactivator-1*α* (PGC-1*α*) signaling pathways enhance anti-tumor immunity mediated by PD-1 blockade

Mitochondria play a central role in energy metabolism. Mitochondria-based lipid metabolism and oxidative phosphorylation are considered crucial in the formation and maintenance of memory T (Tm) cells [78]. Buck et al. reported that mitochondrial morphological changes led to metabolic reprogramming, thereby controlling T cell differentiation. Effector T (Te) cells showed small, distinct mitochondria widely dispersed in the cytoplast whereas Tm cells had densely fused mitochondria. Moreover, forced expression of Opa1, a gene required for mitochondrial fusion, induced Tm cell differentiation, suggesting that mitochondrial metabolism determines T cell differentiation [79]. We showed that mitochondrial activation in tumor-reactive CTLs during PD-1-blocking antibody therapy increased mitochondrial production of reactive oxygen species (ROS) [80]. Furthermore, medication promoting ROS production and metabolism-related substances activating mitochondria enhanced PD-1 blockade cancer immunotherapy. In these models, mammalian target of rapamycin (mTOR) and 5′ adenosine monophosphate-activated protein kinase (AMPK) were both activated in tumor-reactive T cells, and the activation of these related signals by low-molecular weight compounds increased the therapeutic efficacy of PD-1-blocking antibody therapy (Fig. 3). Importantly, peroxisome proliferator-activated receptor (PPAR) gamma coactivator-1*α* (PGC-1α)/PPAR signals, which promote mitochondrial activation, were also activated in the downstream. Tumor inhibitory effects were therefore enhanced by the combination treatment with bezafibrate, a PGC-1*α*/PPAR complex ligand, which has been used for the treatment of hyperlipidemia in the clinic (Fig. 3) [80]. Additionally, a significant increase in total energy metabolism (mitochondrial metabolism and glycolysis) via PGC-1*α*/PPAR signaling was observed in the T cells of mice receiving combination therapy with bezafibrate and PD-1-blocking antibodies. PGC-1*α*/PPAR signaling induced high expression of carnitine palmitoyltransferase 1a (Cpt1a, a fatty acid oxidation-related gene) and B-cell lymphoma 2 (Bcl-2, an apoptosis inhibitory gene), which prevent T cells from activation-induced apoptosis [81]. These systems may convert tumor-reactive Te cells, which are supposed to be short-lived, into long-surviving Te cells, resulting in an overall increase of Te numbers and thus enhancing therapeutic efficacy. Scharping et al*.* reported low PGC-1*α* expression levels in exhausted T cells within a tumor microenvironment, although forced PGC-1*α* expression in T cells rescued cells from a state of exhaustion via mitochondrial-related metabolic reprogramming and enhanced tumor inhibition [82]. Anti-tumor responses may therefore be improved by energy metabolism control via the mitochondria in T cells. The authors also investigated differences in the ability to induce a hypoxic tumor microenvironment, which regulates responsiveness to immune checkpoint inhibitors, in a mouse model. Metformin, which is used to treat type 2 diabetes, suppressed intratumoral oxygen consumption by tumor cells in vitro and in vivo, reduced the intratumoral hypoxic state, and improved intratumoral T cell function, when used in combination with PD-1 antibodies [83]. Bezafibrate and metformin cause fewer side effects and have been used to treat other diseases, suggesting the greater feasibility of combination therapy with these drugs and cancer immunotherapy.

## Future perspectives

PD-1 antibodies have been rapidly developed after their introduction into cancer immunotherapy. However, treatment efficacy varies widely according to cancer type, and treatment cannot be applied uniformly even to patients with the same cancer type. Overall, many challenges remain in the context of PD-1 antibody treatment. Further improvement in cancer care relies on: (1) elucidation of the fundamental mechanisms explaining how PD-1 antibodies possess anti-tumor effects, (2) biomarker development for better prediction of therapeutic and side effects, and (3) the development of combination therapy with fewer side effects. Novel findings and strategies addressing the three issues mentioned here would ensure the improvement of cancer immunotherapy.
